# Living the questions: reflections of a learner in global mental health

**Published:** 2017-04-20

**Authors:** Roselyn Wilson

**Affiliations:** 1 Department of Psychiatry and Behavioural Neurosciences, McMaster University, Ontario, Canada

*Have patience with everything unresolved in your heart and … try to love the questions themselves as if they were locked rooms or books written in a very foreign language. Don’t search for the answers, which could not be given to you now, because you would not be able to live them. And the point is to live everything. Live the questions now. Perhaps then, someday far in the future, you will gradually, without even noticing it, live your way into the answer*.

*– Rainer Maria Rilke*

I entered university brimming with idealism and the desire to help. My premedical studies only partially satisfied this desire. I wanted to go beyond North America and do global health work; my heroes were Paul Farmer and other pioneers of social medicine. Motivated by their examples, I applied for some research funds from my university, and at 21 years of age found myself heading for Kampala, Uganda to gather data for an ambitious qualitative research project, on university students’ attitudes towards prevention of HIV/AIDS. Immediately after I touched down at Entebbe Airport I was confronted with some uncomfortable realities. I noticed the privilege and financial security I enjoyed compared to my Ugandan research subjects – who, as university students, were also my peers. I marveled at the incredible opportunity I had to do cross-cultural research as a mere university student, and realized my audacity in attempting this work despite my relative lack of experience and training. I was also acutely aware of the political and moral implications of my research topic. As I was embarking on this research, the reasons behind the success of Uganda’s HIV/AIDS prevention strategy were being hotly debated by the international academic community, large international funding organizations, and the Ugandan public.^1^ Thoughtful research might bring clarity to this struggle - but was I the person to design and implement it? Most of all, I wondered how my results could possibly be useful to the Ugandan students who so graciously tolerated my research questionnaire and my endless tape recording.

When, two months later, I unpacked my bags back home in Chicago, I found that my uncomfortable questions had travelled home with me as well. Ultimately, these questions left me so tangled in self-doubt and uncertainty that I was unable to finish the research, which then led to a sense of unfilled moral responsibility to my Ugandan friends. I feared that I had done more harm than good – primarily by wasting time and resources better spent elsewhere. I have since realized that my experience was not unique amongst learners from North America travelling to the Global South for research or educational opportunities. Hanson et al.^2^ and Pinto and Upshur^3^ among others have described some of the ethical challenges faced by learners, including the disparity in opportunities between sending institutions and hosts and the possibility that North American students may inadvertently cause harm by creating a burden on their hosts which exceeds their limited ability to be useful.

So I packed my research notebooks away, and shelved my questions as well, apologizing to my Ugandan friends for leaving the project unfinished. I resolved to focus on local work and imagined that answers would come more easily in that setting. Eventually this work led me to medical school. Medical training promised a method of finding answers, through careful empirical observation and application of the best evidence to the patient problem at hand. I learned to search for randomized controlled trials or, better yet, meta-analyses, to aid my clinical decision-making. Yet, I remained perplexed. I encountered many patients that did not experience meaningful improvements in their health despite evidence-based care. Worse, there were many complex patients for whom no evidence seemed to apply.^4^ Behind these examples lurked new questions – who created the evidence, and why? I learned about publication bias and the powerful influence of pharmaceutical and medical device industries^4^,^5^ and even the conceptual limitations of empiricism itself.^6^

My questions did not abate when I began my residency training in psychiatry. I learned many evidence-based interventions for psychotic disorders, but the problem of how to help a chronically homeless, socially isolated person with schizophrenia presenting to the ER was often more complicated than a reductionist meta-analysis could address. It seemed that the empirical evidence, while helpful, was not enough.

Somewhat to my surprise, I found the questions that I faced daily in my practice drawing me yet again to global health. I was intrigued by reports of better outcomes of severe and persistent mental illness in lower/middle-income countries,^7^ and I wondered what we in North America could learn from our colleagues in the Global South. As I wrestled with diagnostic uncertainty in my practice, I encountered literature which questioned the validity of Western diagnostic labels across cultures, and raised concerns that Western psychiatric care may cause significant harm in some settings.^8^ At the same time I read of people with psychotic disorders living in rural villages in eastern Africa, sometimes confined with shackles for their own and others’ safety, who benefited greatly from aspects of Western psychiatric treatment.^9^ Patel,^10^ Kleinman,^11^ and others made compelling moral arguments to provide effective, evidence-based mental health care in lower/middle-income countries. Their voices stirred me, but my previous experience in Uganda filled me with caution. I wondered if global health researchers and clinicians coming from a Western framework could truly avoid the blunders I had inadvertently committed.

Soon after I began residency, our department was invited by colleagues at Mbarara University of Science & Technology (MUST) in western Uganda to support their emerging postgraduate psychiatry program. This partnership, inspired by the educational collaboration between the psychiatry departments of Addis Ababa University and the University of Toronto,^12^ aims to support MUST training of psychiatrists, and ultimately increase the availability and quality of mental health care in western Uganda.

As I became involved in our partnership planning, all of my old questions and misgivings emerged: the fear of doing harm; mistrust of my own motives; concern about reinforcing existing unjust hegemonies. I realized that my questions about global health were intertwined with my questions about psychiatry. I wondered if the questions themselves could be a starting point for further engagement rather than a barrier to participation.

I confess that I am still often confused about how to ethically engage in global health work, and find that over time my questions are only deepening and multiplying. The words of Rilke^13^ comfort me in a way that empiricism cannot, by suggesting an alternative to shutting away difficult questions - living them. For me, living the questions has involved times of direct engagement with global health work, and times of withdrawal and reflection on my experiences and motives.

I am partially driven by a sense of moral responsibility and an awareness of my privilege in having received an expensive and lengthy education. I look to the available global health literature for guidance on ways to do more good than harm.

In the end, however, the most persistent motivators are the same which fuel my clinical work in Canada: stories and relationships. The rich stories of our Ugandan colleagues, of Ugandans living with mental illness, and of our emerging partnership itself are a salve for the painful uncertainty of unanswered questions.
